# Syphilitic proctitis with profuse rectal bleeding in an HIV-infected patient with preserved CD4 count (case report)

**DOI:** 10.1097/MD.0000000000051230

**Published:** 2026-10-30

**Authors:** Mohamad H. Mosi, Hisham Alfarra, Chandni Davi, Shaila Saaki, Refat Jayyusi, Logaen Kubaisy, Hayder Yasir

**Affiliations:** a Department of Internal Medicine, Damascus University Faculty of Medicine, Damascus, Syria; b Department of Internal Medicine, Texas Tech University Health Sciences Center, Amarillo, TX; c Department of Radiology, University of Alabama at Birmingham, Birmingham, AL.

**Keywords:** doxycycline, HIV, syphilitic proctitis

## Abstract

**Rationale:**

Syphilitic proctitis is an uncommon and frequently under-recognized manifestation of *Treponema pallidum* infection that can closely mimic inflammatory, infectious, or neoplastic rectal disease. We report an unusually severe, hemorrhagic presentation in a patient with human immunodeficiency virus (HIV) and a preserved cluster of differentiation 4 (CD4) count, a setting in which overlapping etiologies often delay the diagnosis.

**Patient concerns:**

A heterosexual man in his early 50s with untreated HIV infection and chronic hepatitis B–related cirrhosis (Child-Pugh class B) presented with sudden onset of profuse hematochezia, watery diarrhea, abdominal cramping, and fatigue.

**Diagnoses:**

Computed tomography angiography revealed pronounced rectal wall thickening with deep ulceration. Colonoscopy demonstrated an indurated, ulcerated rectal lesion, and biopsy showed dense lymphoplasmacytic inflammation, with spirochetes confirmed by *T pallidum* immunohistochemistry. Negative cytomegalovirus (CMV) immunohistochemistry, undetectable CMV DNA in plasma and rectal tissue, and a preserved CD4 count (445 cells/µL) made CMV proctitis unlikely. The overall clinical, endoscopic, and histologic findings supported a diagnosis of syphilitic proctitis.

**Interventions:**

Management included doxycycline 100 mg twice daily for 2 weeks, selected because of the patient’s documented penicillin allergy. Antiretroviral therapy with bictegravir/tenofovir alafenamide/emtricitabine was initiated to treat HIV and provide concurrent hepatitis B coverage.

**Outcomes:**

Within 2 weeks, the patient experienced complete resolution of hematochezia and diarrhea. At 3-month follow-up, the CD4 count had risen to 570 cells/µL, HIV RNA was undetectable (<20 copies/mL), and the rapid plasma reagin titer had declined from 1:512 to 1:32.

**Lessons:**

This case underscores that syphilitic proctitis may manifest with severe ulcerative disease even in patients with relatively preserved immune function. Clinicians should maintain vigilance for syphilis in individuals with HIV who present with rectal bleeding or proctitis to prevent diagnostic delays and ensure timely therapy.

## 1. Introduction

Syphilis, primarily caused by *Treponema pallidum*, is known to involve the genitals, the central nervous system (CNS), and skin. However, on rare occasions, it has been reported to affect the gastrointestinal system, presenting with dyschezia, hematochezia, tenesmus, altered bowel habits, and mucus in stool, which could be easily misdiagnosed. In particular, syphilitic proctitis has been seldom reported, but with the recent increase in the incidence of syphilis among men who have sex with men (MSM) and heterosexuals, it is gaining recognition.^[1]^ In a review study, out of all cases of proctitis in MSM, 0.05% are due to syphilis, presenting with commonplace rectal symptoms.^[2]^

Primary syphilis presents within 3 months after inoculation as a painless chancre and is often missed, but sometimes these anorectal chancres may be associated with itching, bleeding, and tenesmus. Primary syphilitic lesions may be confused with or coexist with other sexually transmitted infections or opportunistic infections in human immunodeficiency virus (HIV) patients. Secondary syphilitic lesions, condyloma latum, may resemble human papillomavirus warts and could present with a widespread maculopapular rash and mucous membrane lesions, including track ulcers or mucous patches, which may affect the rectal lining. These lesions, both primary and secondary, are highly infectious due to a high treponeme burden.^[2]^

Syphilis testing includes the following: detection of antibodies in serum; visualization of the organism using darkfield or fluorescent microscopy on tissue samples from lesions; analysis of infected tissue under microscopy; or detection of *T pallidum* nucleic acid-specific sequences in samples, including fluids, exudates, or tissue biopsies. In addition, testing for other sexually transmitted diseases should also be performed.

This case report aims to characterize a rare condition observed in individuals living with HIV, occurring across a spectrum of immunosuppression severity. The report outlines the clinical presentation, diagnostic evaluation, and therapeutic considerations, while integrating a focused discussion of the key differential diagnoses relevant to this entity.

## 2. Case presentation

An early 50s heterosexual male with a history of hypertension, untreated human immunodeficiency virus (HIV) infection due to poor outpatient compliance, chronic hepatitis B, and cirrhosis (Child-Pugh class B, 7 points; no ascites or hepatic encephalopathy) presented to the emergency department with profuse rectal bleeding, watery bloody diarrhea averaging 10 episodes per day, fatigue, and abdominal cramping of 28 hours’ duration. He reported prior alcohol use discontinued 12 years earlier and denied tobacco use. On admission, vital signs were stable (blood pressure, 124/75 mm Hg; heart rate, 95 beats per minute; oxygen saturation, 98% on room air), and physical examination revealed gross blood on digital rectal examination.

Computed tomography (CT) angiography demonstrated marked rectal wall thickening with perirectal stranding consistent with severe colitis/proctitis, mild mucosal enhancement suggestive of intraluminal blood products, cirrhosis with portal hypertension, and bladder wall thickening, without evidence of focal contrast extravasation (Fig. 1).

**Figure 1.**
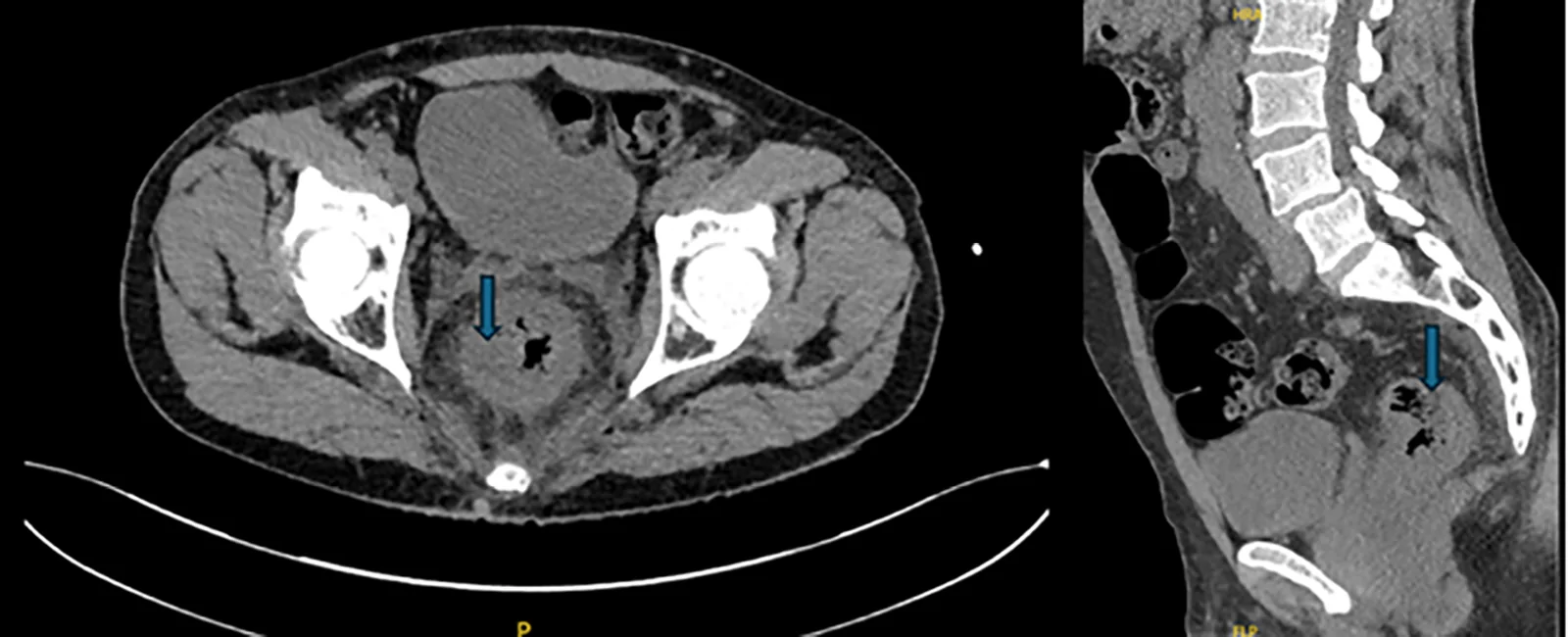
Computed tomography angiography of the abdomen and pelvis demonstrates marked rectal wall thickening on the axial image (blue arrow, left panel) and a focal rectal ulcer visualized on the sagittal reconstruction (blue arrow, right panel).

Laboratory evaluation revealed anemia, thrombocytopenia, hypoalbuminemia, and iron deficiency (Table 1), with infectious workup summarized in Table 2.

**Table 1:** Laboratory values on admission.

Test	Result	Unit
**Hematology**		
White blood cell count (WBC)	3.2	×10^3^/µL
Hemoglobin	8.2	g/dL
Hematocrit	24	%
Platelets	118	×10^3^/µL
**Chemistry and electrolytes**		
Sodium	138	mmol/L
Potassium	3.9	mmol/L
Calcium	8.2	mg/dL
Magnesium	1.2	mg/dL
Glucose	180	mg/dL
Blood urea nitrogen (BUN)	19	mg/dL
Creatinine	1.1	mg/dL
Lactic acid	2.0	mmol/L
**Liver function tests**		
Aspartate aminotransferase (AST)	17	U/L
Alanine aminotransferase (ALT)	24	U/L
Alkaline phosphatase (ALP)	109	U/L
Total bilirubin	1.2	mg/dL
Albumin	2.2	g/dL
**Iron studies and vitamins**		
Serum iron	18	µg/dL
Total iron-binding capacity (TIBC)	229	µg/dL
Transferrin saturation	9	%
Ferritin	29	ng/mL
Vitamin B12	393	pg/mL
**Coagulation**		
Prothrombin time (PT)	11.4	seconds
International normalized ratio (INR)	1.2	—
**Urinalysis**		
White blood cells	0–5	/HPF
Red blood cells	0–2	/HPF
Protein	Positive	—
Nitrite	Positive	—
Bacteria	Trace	—

**Table 2:** Infectious disease and immunologic workup.

Test	Result
**Viral hepatitis**	
Hepatitis A IgM antibody	Negative
Hepatitis B core antibody, total	Positive
Hepatitis B core IgM antibody	Negative
Hepatitis B surface antigen	Positive
**HIV**	
HIV-1/2 antibody	Positive
HIV RNA (plasma)	6020 copies/mL
Absolute CD4 count	445 cells/µL
**Syphilis**	
RPR titer	1:512
*Treponema pallidum* antibodies	Reactive
FTA-ABS	1:10
**Other infectious workup**	
CMV DNA, plasma (quantitative real-time PCR)	<100 IU/mL (not detected)
CMV DNA, rectal tissue (qualitative PCR)	Negative
*Toxoplasma* IgM antibody	<3 (negative)
Cryptococcal antigen (lateral flow assay)	Negative
QuantiFERON-TB Gold Plus	Negative

Plasma CMV DNA was measured by quantitative real-time PCR; a result of <100 IU/mL indicates that CMV DNA was not detected. Cryptococcal antigen was tested by lateral flow assay.

CMV = cytomegalovirus, FTA-ABS = fluorescent treponemal antibody absorption, HIV = human immunodeficiency virus, IgM = immunoglobulin M, PCR = polymerase chain reaction, RPR = rapid plasma reagin.

Esophagogastroduodenoscopy identified grade 1 esophageal varices without active bleeding, while colonoscopy revealed an indurated, ulcerated lesion in the mid-distal rectum extending toward the anal verge with deep ulceration (Fig. 2).

**Figure 2.**
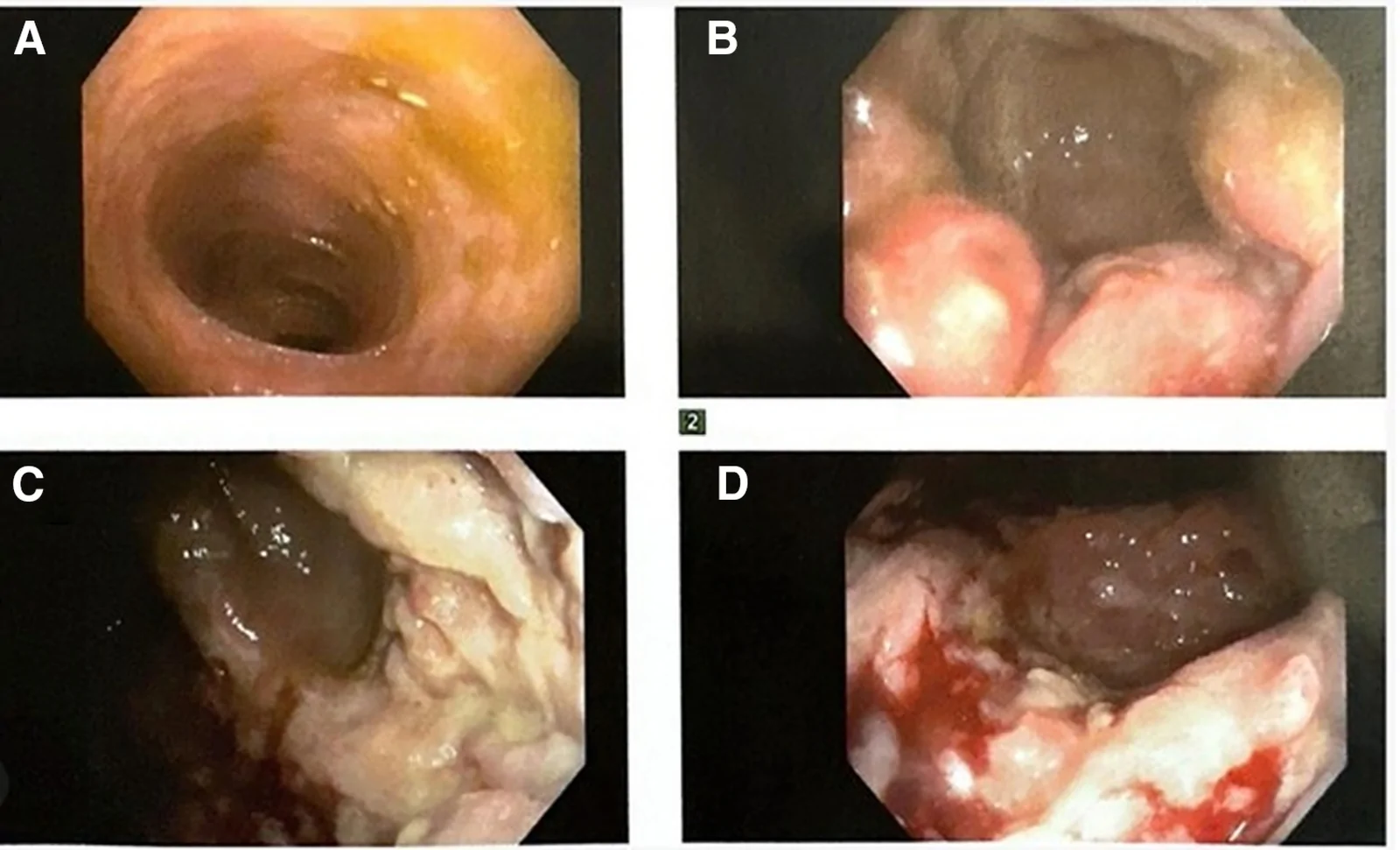
Colonoscopy findings. (A) Proximal rectum showing erythematous mucosa with yellowish exudate and a preserved lumen. (B) Nodular, indurated lesion with ulceration in the mid-distal rectum. (C, D) Deep ulceration with fibrinous exudate, necrotic-appearing debris, and contact bleeding extending toward the anal verge.

Histopathological examination of rectal biopsies demonstrated predominantly granulation tissue with marked mixed inflammatory infiltrates, ulceration with features of both active and chronic inflammation, and dense lymphoplasmacytic inflammation (Fig. 3A). Immunohistochemical staining with a polyclonal anti–*T pallidum* antibody demonstrated numerous spirochetes (Fig. 3B), whereas immunohistochemical stains for cytomegalovirus (CMV) and herpes simplex virus were negative. Taken together, the clinical, endoscopic, and histologic findings raised suspicion for an infectious etiology, with syphilitic proctitis considered in the differential diagnosis.

**Figure 3.**
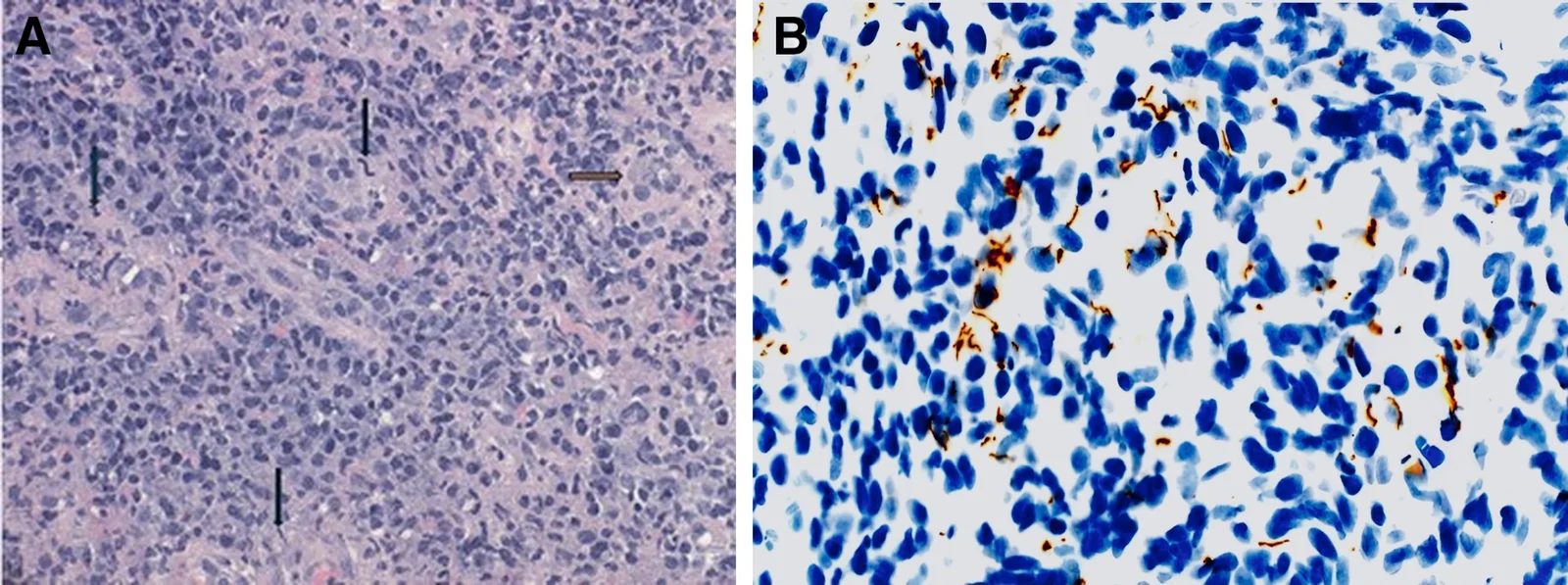
(A) Rectal ulcer biopsy demonstrating predominantly granulation tissue (horizontal arrow) accompanied by a marked mixed inflammatory infiltrate with dense lymphoplasmacytic inflammation (vertical arrows); hematoxylin and eosin, original magnification ×300 (oil immersion). (B) Immunohistochemical stain with a polyclonal anti–*Treponema pallidum* antibody demonstrating numerous spirochetes (brown chromogen); high-power view.

Given the patient’s cluster of differentiation 4 (CD4) count of 445 cells/µL, plasma CMV DNA below the limit of detection on quantitative real-time polymerase chain reaction (PCR; <100 IU/mL, not detected), a negative qualitative CMV DNA PCR on rectal tissue, and negative CMV immunostaining on rectal biopsy, CMV proctitis was considered unlikely. The ulcerative lesions were therefore attributed to syphilitic proctitis as the most plausible diagnosis. To exclude neurosyphilis, a lumbar puncture was performed, and cerebrospinal fluid analysis – including Venereal Disease Research Laboratory testing, rapid plasma reagin, white blood cell count, and protein – was within normal limits.

Although intramuscular benzathine penicillin G remains the standard therapy for syphilitic proctitis, doxycycline is an established alternative in patients with penicillin allergy.^[3]^ Because our patient had a documented penicillin allergy, he was treated with oral doxycycline 100 mg twice daily for 2 weeks, the guideline-recommended alternative regimen for early syphilis.

Given the patient’s HIV infection and concurrent hepatitis B surface antigen positivity, antiretroviral therapy was initiated with the fixed-dose combination of bictegravir/tenofovir alafenamide/emtricitabine, providing a fully suppressive regimen with dual activity against both HIV and hepatitis B virus.

The patient experienced complete resolution of hematochezia and diarrhea within 2 weeks. He was discharged with outpatient follow-up for repeat RPR titers, HIV viral load monitoring, and hepatology evaluation. At 3-month follow-up, he reported adherence to antiretroviral therapy and remained free of hematochezia, diarrhea, and abdominal pain, and a repeat complete blood count had normalized. The CD4 count increased to 570 cells/µL, HIV RNA was undetectable (<20 copies/mL), and the RPR titer declined from 1:512 to 1:32, consistent with an adequate serologic response. Repeat endoscopy was not performed because it was not clinically indicated in the setting of complete clinical and serologic resolution.

## 3. Discussion

Syphilitic proctitis represents a relatively rare clinical manifestation of secondary *T pallidum* infection, most commonly observed among MSM and individuals with concomitant HIV infection. In immunocompromised hosts, particularly those with advanced HIV disease, the infection may progress rapidly and present with nonspecific anorectal complaints, including pain, tenesmus, purulent discharge, or rectal bleeding. Notably, up to one third of affected patients may remain asymptomatic, further hindering timely recognition and diagnosis.^[4]^ Our patient, by contrast, was heterosexual and had a known diagnosis of HIV. He presented with an unusually acute and severe clinical picture, including profuse rectal hemorrhage, bloody watery diarrhea, marked fatigue, and abdominal cramping.

Cardona Arias et al reported a 0.6% coinfection prevalence of *T pallidum* and HIV in their cohort.^[5]^ Notably, syphilitic proctitis has been reported both in individuals with profound immunosuppression, particularly those with CD4 counts below 50 cells/µL, as well as in patients with more moderate immunosuppression, including cases with CD4 counts 227 and 275 cells/µL.^[6–8]^ In contrast, the patient in our case exhibited clinical features consistent with syphilitic proctitis despite having only mild immunosuppression (CD4 = 445 cells/µL) and not being on antiretroviral therapy.

Diagnosis of syphilitic proctitis relies on the integration of clinical presentation, imaging characteristics, endoscopic evaluation with histopathology, and syphilis serology. On cross-sectional imaging, contrast-enhanced CT typically demonstrates features of inflammatory proctitis, most notably concentric rectal wall thickening and perirectal fat stranding. Some reports describe a “target sign,” reflecting smooth, circumferential mural thickening with layered enhancement patterns.^[4]^ In our case, contrast-enhanced CT revealed rectal wall thickening measuring approximately 9 mm, accompanied by an ulcerated segment best appreciated on sagittal reconstructions (Fig. 1).

Histopathologically, syphilitic proctitis often demonstrates a plasma-cell–rich inflammatory infiltrate, nonspecific chronic mucosal injury, and, in some cases, direct visualization of spirochetes on earlier biopsies.^[9,10]^

In our case, the rectal biopsy revealed predominantly granulation tissue with a marked mixed inflammatory infiltrate, accompanied by ulceration showing features of both active and chronic inflammation. Notably, there was dense lymphoplasmacytic inflammation on hematoxylin and eosin staining, and immunohistochemistry with a polyclonal anti–*T pallidum* antibody demonstrated abundant spirochetes, supporting the diagnosis of syphilitic proctitis (Fig. 3).

One of the key differential diagnoses for syphilitic proctitis is cytomegalovirus (CMV) proctitis, an opportunistic infection most commonly encountered in individuals with significant immunosuppression, particularly those with advanced HIV infection or profound CD4 depletion (often CD4 < 50 cells/µL). Although classically associated with severe immunosuppression, CMV proctitis could occur in immunocompetent individuals. Clinically, it presents with rectal pain, tenesmus, diarrhea, and hematochezia – a constellation of symptoms that closely overlaps with syphilitic proctitis.

Endoscopically, CMV proctitis frequently demonstrates mucosal ulcerations, and histopathologic evaluation remains the diagnostic gold standard. Biopsies typically reveal cytomegalic cells with characteristic intranuclear and intracytoplasmic (“owl’s eye”) inclusions, accompanied by granulation tissue and a mixed inflammatory infiltrate. Immunohistochemistry for CMV antigen further enhances diagnostic sensitivity.^[11]^

In our case, the patient’s symptoms were clinically compatible with either syphilitic or CMV proctitis. However, the absence of CMV immunostaining on biopsy, an undetectable plasma CMV DNA level (<100 IU/mL), and a negative qualitative CMV DNA PCR on rectal tissue make CMV proctitis highly unlikely, supporting syphilis as the more plausible etiology. Taken together, the reactive RPR at a titer of 1:512 with confirmatory treponemal testing, immunohistochemically confirmed spirochetes in the rectal biopsy, exclusion of CMV across 3 independent modalities, and the rapid clinical and serologic response to therapy provide convergent evidence for syphilitic proctitis.

Management of syphilitic proctitis has most commonly involved intramuscular benzathine penicillin G, which remains the standard therapeutic regimen. Carmona Valencia et al^[4]^ reported successful treatment using 3 weekly doses of 2.4 million IU, whereas Díaz Jaime et al^[9]^ described resolution with a single 2.4 million IU dose in cases of early syphilis. In contrast, Hunter et al^[10]^ documented effective management with oral doxycycline, underscoring its role as an alternative option when penicillin is contraindicated or unavailable.^[3]^

In our case, the patient was initiated on oral doxycycline, resulting in marked improvement in rectal pain, hematochezia, and overall gastrointestinal symptoms. This favorable clinical response is consistent with prior reports supporting doxycycline.

## 4. Learning points

Syphilitic proctitis can cause severe ulceration and bleeding in heterosexual patients even with mild HIV-related immunosuppression.

CMV and syphilitic proctitis are differential diagnoses for rectal ulceration.

Doxycycline is an effective alternative therapy when penicillin cannot be used, such as in patients with penicillin allergy.

## Author contributions

**Conceptualization:** Mohamad H. Mosi, Refat Jayyusi.

**Data curation:** Mohamad H. Mosi, Chandni Davi, Refat Jayyusi.

**Project administration:** Chandni Davi.

**Validation:** Chandni Davi.

**Formal analysis:** Shaila Saaki.

**Funding acquisition:** Shaila Saaki.

**Visualization:** Shaila Saaki.

**Investigation:** Logaen Kubaisy.

**Resources:** Logaen Kubaisy.

**Supervision:** Hayder Yasir.

**Writing – original draft:** Mohamad H. Mosi, Shaila Saaki, Refat Jayyusi.

**Writing – review & editing:** Mohamad H. Mosi, Hisham Alfarra.
